# Genome Sequence of a Fish-Associated Polyomavirus, Black Sea Bass (Centropristis striata) Polyomavirus 1

**DOI:** 10.1128/genomeA.01476-14

**Published:** 2015-01-29

**Authors:** Alberto Peretti, Peter C. FitzGerald, Valery Bliskovsky, Diana V. Pastrana, Christopher B. Buck

**Affiliations:** National Cancer Institute, Bethesda, Maryland, USA

## Abstract

All known polyomaviruses are associated with mammals or birds. Using virion enrichment, random-primed rolling circle amplification, and deep sequencing, we identified a polyomavirus associated with black sea bass (Centropristis striata). The virus has a variety of novel genetic features, suggesting a long evolutionary separation from polyomaviruses of terrestrial animals.

## GENOME ANNOUNCEMENT

Members of the circular-DNA viral family *Polyomaviridae* have so far been conclusively documented only in mammals and birds (1, 2). In this genome announcement, we report the sequence of a complete polyomavirus genome isolated from a marine fish. A black sea bass (Centropristis striata) was purchased from a supermarket near Bethesda, Maryland. Roughly 3 g of tissue was collected from the scales, skin, lateral muscle, mouth, eye, anal/genital opening, swim bladder, and liver. The mixed-tissue sample was subjected to virion enrichment and deep sequencing, as previously described (3). BlastX searches revealed a ~300 bp overlapping readpair with about 35% homology to the zinc-binding domains of various polyomavirus large T antigen (LT) proteins. Another readpair with homology to LT ATPase domains was also observed. PCRs using outward-directed primers were performed targeting each read pair. The PCR products were gel purified, cloned, and sequenced by primer walking. The two clones showed nearly identical sequences, with only minor variations in the length of poly-T tracts in the protein-noncoding regulatory region.

The complete circular genome of black sea bass-associated polyomavirus 1 (BassPyV1) is 7.4 kb in length. This is substantially larger than previously described polyomaviruses, which range from 4.7 to 5.7 kb in length (4, 5).

The predicted LT protein of BassPyV1 is encoded by a single large open reading frame (ORF). Its closest homolog is the LT protein of a chimeric virus known as Japanese eel endothelial cells-infecting virus (JEECV) (6). BassPyV1 LT carries a classic pRb-binding (LXCXE) motif, a possible conserved region 1 (CR1), recognizable origin-binding, zinc finger, and ATPase domains, and a C-terminal F-Box/WD repeat-containing protein 7 (Fbw7)-binding motif (7). The encoded protein lacks other familiar LT features, such as the DNAJ and Bub1 motifs. Like JEECV, the BassPyV1 early region does not appear to encode a small t antigen.

At 599 amino acids in length, the predicted VP2 minor capsid protein of BassPyV1 is about twice as long as typical VP2 proteins. Like other VP2 proteins, the BassPyV1 VP2 encodes a possible myristoylation motif at its N terminus. There does not appear to be a VP3 homolog encoded within BassPyV1 VP2 (8).

The predicted VP1 major capsid protein ORF is of typical length (353 amino acids). Analysis of potential splicing signals suggests that the virus may express an unusual N-extended form of VP1 from a spliced mRNA. The hypothetical first exon of VP1 encodes a cysteine-rich leader analogous to flexible N-terminal motifs that are known to form stabilizing disulfide bonds in the virions of other polyomavirus species (9).

Although it is theoretically possible that BassPyV1 represents an environmental contaminant (as opposed to actual infection of the fish itself), phylogenetic analysis of its sequence indicates that it is extremely divergent from all previously described terrestrial polyomaviruses. A possible explanation for this divergence is that BassPyV1 evolved separately during the >350 million years since marine and terrestrial animals last shared a common ancestor.

### Nucleotide sequence accession number.

The complete genomic sequence of black sea bass polyomavirus 1 was deposited in GenBank under the accession number KP071318.
